# Supplementary material for: Characterization of Stem Nodes Associated with Carbon Partitioning in Maize in Response to Nitrogen Availability
Source: Int J Mol Sci. 2022 Apr 15;23(8):4389. doi: 10.3390/ijms23084389 (PMC9024680; doi:10.3390/ijms23084389)
Supplement: Supplementary file 1 [file ijms-23-04389-s001.zip › ijms-1670752-supplementary.pdf]

Supplementary Table S1. Effects of nitrogen (N) availability on maize yield and its components at maturity[1].

N treatment	Grain yield (g plant^{-1})	Row number per cob	Kernel number per row	Kernel weight (g kernel^{-1})
**LN**	9.11±4.71 b	5.3±1.8 b	5.06±1.78 b	1.24±1.10 a
**MN**	66.10±9.99 a	13.3±0.8 a	19.50±2.59 a	0.25±0.01 b
**HN**	87.65±2.24 a	14.7±0.8 a	22.89±1.44 a	0.26±0.00 b

[1] Data are mean ± standard error (n = 4). Means with no letter in common indicate significant differences between N treatment for each parameter ($p < 0.05$). LN, low N; MN, medium N; HN, high N.

Supplementary Table S2. Effects of nitrogen (N) availability on the concentrations of soluble sugars (sum of fructose, glucose, and sucrose) in leaves and stem nodes at 62 days after sowing[1].

**N treatment**	**Sugar concentrations in different tissues (mg g^{-1})**				
	8th leaf	Node 1	Node 2	Node 3	Node 8
**LN**	19.11±1.92 b	65.14±7.78 b	46.64±4.38 b	52.25±4.59 a	35.95±2.59 b
**MN**	46.60±2.17 a	141.01±4.95 a	64.97±5.60 a	60.14±7.13 a	64.80±2.20 a
**HN**	49.12±6.97 a	123.40±6.11 a	63.60±5.41 a	50.86±9.05 a	67.47±5.43 a

[1] Data are mean ± standard error (n = 4). Means with no letter in common indicate significant differences between N treatment for each parameter ($p < 0.05$). LN, no nitrogen application; MN, 0.15 g N kg^{-1} soil; HN, 0.30 g N kg^{-1} soil.

Supplementary Table S3. List of primers used in this research.

Gene	Primer sequence (5'-3')
ZmSWEET3a (GRMZM2G179679)	Fw: CATTTACAGCAGATGCAATCGA
	Re: AGGCAGTTTCTTAAGACTCTCC
ZmSWEET4a (GRMZM2G000812)	Fw: TGTACGCCATCTACTACAAGTC
	Re: ATATTGGTGTCGATCGTCACTT
ZmSWEET4b (GRMZM2G144581)	Fw: AACTGTGCTCAATCGTTTCTTC
	Re: GAGAGTGTGCTATTTTGCTAGC
ZmSWEET13a (GRMZM2G173669)	Fw: TGTGTTGGGAAATTCTTGTGAC
	Re: GATGACGTTACCTAGTAGACCG
ZmSWEET13b (GRMZM2G021706)	Fw: CATCATACAACACCAGCAACTG
	Re: CGACACAAAAAGGGTGATACAG
ZmSWEET13c (GRMZM2G179349)	Fw: GTCATCTCCTTCATGACCTTCC
	Re: GAAGTACATGACCACGTAGACG
ZmSUT1 (GRMZM2G034302)	Fw: GGCACAAGTGGTTTCCGTTC
	Re: TTTGCCTTTGTGGGGAGGTT
ZmSUT2 (GRMZM2G307561)	Fw: TCCATCCACTCTAGGTCTCTAG
	Re: GAATCGAATCGCTGAGTTGTTT
ZmSUT4 (GRMZM2G145107)	Fw: AACATTTTGACCAGAATGAGGC
	Re: ATTTGGATCCCCATGGTAAACT
ZmTublin	Fw: GCTATCCTGTGATCTGCCCTGA
	Re: CGCCAAACTTAATAACCCAGTA

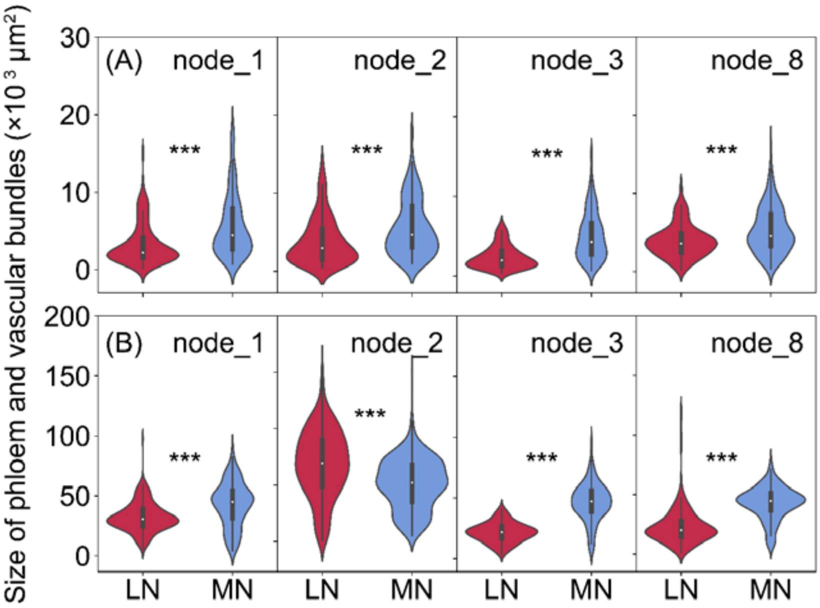

Supplementary Figure S1. The size of phloem (A to D) and vascular bundles (E to H) in the internodes (2 cm above node_1, node_2, node_3, and node_8 of maize plants at 62 days after sowing under low and medium N supply. The solid line and dash line within each box represent the median and mean values of all data. The top and bottom edges represent the 75 and 25 percentiles, and the top and bottom bars represent the 95 and 5 percentiles of all data, respectively. ***, $p < 0.001$.

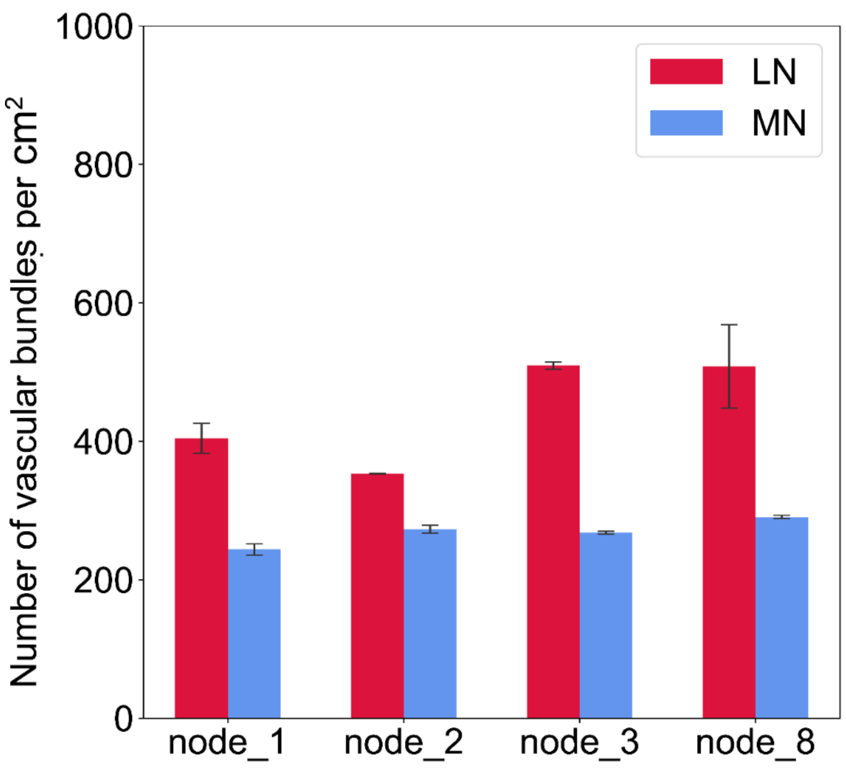

Supplementary Figure S2. The number of vascular bundles per unit area in the internodes (2 cm above node_1, node_2, node_3, and node_8 of maize plants at 62 days after sowing under low and medium N supply.
